# Evaluating the Risks of Heated Tobacco Products: Toxicological Effects on Two Selected Respiratory Bacteria and Human Lung Cells

**DOI:** 10.3390/toxics13020070

**Published:** 2025-01-21

**Authors:** Salvatore Furnari, Rosalia Emma, Massimo Caruso, Pio Maria Furneri, Virginia Fuochi

**Affiliations:** 1 Department of Biomedical and Biotechnological Sciences (BIOMETEC), University of Catania, 95124 Catania, Italymassimo.caruso@unict.it (M.C.); furneri@unict.it (P.M.F.); 2 Center of Excellence for the Acceleration of Harm Reduction (CoEHAR), University of Catania, 95124 Catania, Italy; 3 Department of Clinical and Experimental Medicine (MEDCLIN), University of Catania, 95124 Catania, Italy

**Keywords:** tobacco heating products (THPs), *Streptococcus pneumoniae*, *Klebsiella pneumoniae*, lung health, respiratory microbial communities, cytotoxicity, mutagenicity

## Abstract

Heated tobacco products (THPs) are increasingly promoted as potential harm reduction tools, offering an alternative to traditional cigarettes. Despite these claims, understanding of their toxicological impact on respiratory health and associated microbial communities is limited. Comprehensive investigations are needed to elucidate the biological mechanisms and potential health implications associated with their use. Methods: This study evaluated the toxicological effects of aerosols produced by THPs (IQOS 3 Duo with Heets “Sienna Selection”) in comparison to conventional cigarette smoke (1R6F). Antibacterial activity was evaluated using *Streptococcus pneumoniae* and *Klebsiella pneumoniae* as representative species of the respiratory microbiota through agar diffusion assays and MIC/MBC determinations. Cytotoxicity was assessed in human lung fibroblast cells (MRC5) through the neutral red uptake (NRU) assay, whereas mutagenicity was investigated using the Ames test. Results: THP aerosols demonstrated the ability to inhibit the growth of both *S. pneumoniae* and *K. pneumoniae*, exerting bacteriostatic effects at lower concentrations and bactericidal effects at higher concentrations. While these antibacterial effects might initially seem beneficial against pathogens such as *K. pneumoniae*, they raise concerns about the potential disruption of the respiratory microbial balance, particularly in relation to *S. pneumoniae*. Despite these microbiological effects, THP aerosols demonstrated minimal cytotoxicity on human lung fibroblasts and lacked detectable mutagenic activity, contrasting with the significant cytotoxicity and mutagenicity caused by cigarette smoke. Conclusions: THPs present a reduced short-term toxicological profile compared with conventional cigarettes; however, their effects on respiratory microorganisms deserve attention. The observed inhibition of commensal bacteria highlights the need to explore potential changes in the microbial ecosystem that could affect respiratory health. These findings highlight the need for additional studies to evaluate the long-term effect of THP use on respiratory microbiota and the stability of the overall microbial ecosystem.

## 1. Introduction

Over the past few years, the spreading of heated tobacco products (THPs) has set off significant debate in public health, as these devices are frequently reported as a potentially safer alternative to traditional cigarettes [1,2,3]. Unlike traditional cigarettes, THPs heat tobacco without triggering combustion, thereby minimizing the production of many harmful and potentially harmful substances, including tar and carbon monoxide [4,5]. This has led to the suggestion that THPs may pose fewer risks to human health, particularly in relation to respiratory and cardiovascular diseases [6,7]. However, the reduced-risk claim is not unequivocally supported by evidence, as the long-term biological and public health impacts of THPs remain poorly understood [8].

Among the advantages of using THPs instead of classic cigarettes is the fact that THP companies highlight the potential of THPs to reduce exposure to toxicants, and additionally, they are often marketed as tools for harm reduction, targeting smokers unable or unwilling to quite nicotine use entirely [9]. Despite this, critics argue that THPs are far from risk-free [10]. Actually, many research suggest that these products can still deliver nicotine and other harmful substances at levels capable of inducing adverse effects, including oxidative stress, inflammation, and impaired immune response [11]. Moreover, the aerosol produced by THPs may include unique toxicological profiles distinct from traditional cigarette smoke, with unknown implications for lung health and systemic diseases [12].

Recent research has shifted focus from the chemical analysis of THPs to exploring their biological effects, particularly their interactions with microbial communities. A notable area of investigation is the influence of THP aerosols on bacteria, which are integral to the microbial ecology of environments such as the human respiratory tract. This line of inquiry is important for several reasons. The respiratory tract contains a diverse array of bacteria that contribute to physiological functions and help maintain microbial balance [13]. Disruptions to this balance have been associated with various respiratory conditions, highlighting the need to study how THPs may affect these communities [14,15]. Evaluating the effects of THP aerosols on both pathogenic and commensal microbiota could provide valuable insights into their broader impact on respiratory health [16].

One of the key components of the resident microbiota is *Streptococcus pneumoniae*, a common commensal of the nasopharynx in healthy individuals that plays a significant role in the respiratory microbiota [17,18]. However, under dysbiotic conditions, *S. pneumoniae* can transition from a benign resident to a pathogen, capable of causing invasive diseases such as pneumonia, sepsis, and meningitis [19,20]. Similarly, *Klebsiella pneumoniae*, another opportunistic pathogen, is part of the normal microbiota of the upper respiratory tract and gastrointestinal system but can become a major cause of hospital- and community-acquired infections, including pneumonia and bloodstream infections, particularly under conditions of microbial imbalance [21,22,23]. Smoking has been identified as a major risk factor for diseases associated with both *S. pneumoniae* and *K. pneumoniae*, largely due to its ability to impair mucociliary clearance, modulate immune responses, and enhance bacterial adherence to epithelial cells [24]. Cigarette smoke, for example, alters the microbial landscape of the nasopharynx and lower airways, creating conditions favorable for the colonization and overgrowth of opportunistic pathogens such as *S. pneumoniae* and *K. pneumoniae* [25]. While THPs emit aerosols with a different toxicological profile, their impact on the respiratory microbiota and potential to promote similar pathogenic transitions remains an open question. In the context of THP use, the extent to which these products contribute to dysbiosis and enhance the pathogenic potential of bacteria such as *S. pneumoniae* and *K. pneumoniae* remains underexplored. Investigating these interactions is crucial to understanding whether THPs truly represent a safer alternative to conventional cigarettes or pose distinct risks to respiratory health [26]. Explorations into these dynamics are essential for shaping evidence-based regulatory policies and public health recommendations regarding THP use.

The main objective of this study was to investigate the biological effects of aerosols generated by THPs, with a particular focus on their effects on the proliferation of two bacterial models—one Gram-positive and one Gram-negative—driven by their respective roles as major agents of bacterial pneumonia. Specifically, we aimed to determine whether the proliferation of *S. pneumoniae* and *K. pneumoniae* is altered in the presence of THP aerosols, as this could indicate potential impacts on respiratory health and inflammation compared to traditional cigarettes.

To address these questions, we utilized a combination of quantitative and qualitative assays to specifically evaluate the cytotoxicity, mutagenicity and antimicrobial effects of aerosols generated by the IQOS 3 Duo device with Heets “Sienna Selection” in comparison to the biological activity of traditional tobacco smoke produced by 1R6F Kentucky reference cigarettes. Additionally, an Ames test was conducted to evaluate the mutagenic potential of the aerosol, providing insights into genotoxic risks. Human lung fibroblast diploid cells (MRC-5) were also exposed to cigarette smoke and IQOS vapors to investigate cytotoxic effects on lung tissue. These experiments were designed to offer a thorough evaluation of the relative safety of THPs, focusing on their effects on microbial communities and cellular responses.

## 2. Materials and Methods

### 2.1. Bacterial Strain

*Streptococcus pneumoniae* ATCC 49619 and *Klebsiella pneumoniae* ATCC 700603 strains were used for our purpose (collection of the Laboratory of Applied Microbiology, Department of Biomedical and Biotechnological Sciences, Università di Catania).

### 2.2. Smoke and Aerosol Generation and Aqueous Extracts (AqExs) Production

AqExs were generated by bubbling whole cigarette smoke or THP vapors through 40 mL of CAMHB medium (Cationic Adjusted Mueller Hinton Broth; Oxoid, UK) using a glass impinger trap. 1R6F reference cigarettes (Center for Tobacco Reference Products, University of Kentucky) were smoked using the LM1 smoking machine (Borgwaldt KC GmbH, Hamburg, Germany), while the THPs were vaped using the LM4E vaping machine (Borgwaldt KC GmbH, Hamburg, Germany). The IQOS 3 Duo (Philip Morris Products SA, Lausanne, Switzerland) equipped with Heets “Sienna Selection” was vaped following the Health Canada Intense (HCI) regimen (55 mL puff volume, 3 s puff duration, 30 s inter-puff interval, bell-shaped profile) without blocking the ventilation holes, for a total of 120 puffs (10 Heets × 12 puffs). Moreover, six 1R6F tobacco reference cigarettes (University of Kentucky) were smoked following the HCI regimen (55 mL puff volume, 3 s puff duration, 30 s inter-puff interval, bell-shaped profile) with blocked ventilation holes, for a total of 48 puffs (6 cigarettes × 8 puffs). The aliquots of bubbled media were filtered through a 0.22 µm filter (Millex^®^ Filter Units with MF-Millipore™ Membrane, Sigma-Aldrich, Merck, Darmstadt, Germany) before carrying out subsequent assays to eliminate any contamination introduced during the bubbling process. Furthermore, pH measurements were taken both before and after exposure to smoke/vapors using a pH meter (Eutech pH 700, Eutech Instruments, ThermoFisher, Milan, Italy), which had been previously standardized at room temperature (23 °C). As part of the control procedure, the pH of sterile distilled water was also measured.

### 2.3. Agar Diffusion Test

The antibacterial activity of pure AqExs was assessed using the agar diffusion test, following the method described previously [27,28]. In brief, 100 µL of each sample was dispensed into blank discs in pre-inoculated plates (1.5 × 10^5^ CFU mL^−1^) of Antibiotic Agar Medium No.1 (AAM1; Sigma-Aldrich-Merck KGaA, Darmstadt, Germany), with 5% sheep blood for *Streptococcus*. Phosphate-Buffer Solution (PBS, 100 µL) served as negative control, while Ceftriaxone 30 µg (CRO) was used as positive control. Plates were incubated overnight at 37 °C under aerobic conditions for *Klebsiella* and in a 5% CO_2_ atmosphere for *Streptococcus*. The inhibition zone diameters were measured in millimeters using a caliper, with a precision of 0.1 mm. Each determination was performed in triplicate within the same experiment, and the experiment was repeated three times. Results were expressed as mean ± SD.

### 2.4. Minimum Inhibitory Concentration (MIC) and Minimum Bactericidal Concentration (MBC)

The assays were performed using 96-well polystyrene plates (Corning^®^ 96 Well Microplates, Leipzig, Germany) with the two CAMHB AqExs:

- CAMHB bubbled with tobacco cigarette smoke;
- CAMHB bubbled with IQOS vapors.

According to the CLSI M100 guidelines, MIC values were determined using the microdilution method [28]. The *inocula* were prepared from bacterial suspensions at a 0.5 McFarland standard, and dilutions in untreated broth were made to obtain final concentrations of 10^4^–10^5^ CFU mL^−1^. The plates were incubated overnight at 37 °C under aerobic conditions. The tested concentration range of the bubbled media was from 100% to 6.25% *v*/*v*.

A negative control was performed using CAMHB bubbled with air only, ensuring no antibacterial activity from the medium itself.

The following day, MBC assays were conducted. A total of 10 µL of the dilutions corresponding to the MIC values, along with two more concentrated dilutions, was plated on AAM1 plates (with 5% of sheep blood for Streptococcus). After overnight incubation at 37 °C, the plates were examined, and viable CFU mL^−1^ was enumerated to determine the bactericidal activity.

Each determination was performed in six replicates within the same experiment, and the experiment was repeated six times. Results were expressed as the mode obtained above 50%.

### 2.5. Cell Cultures

The MRC-5 cell line (Lung Normal Fibroblast Cells, ATCC^®^ CCL-171™) was used for this study. The cells were cultured in DMEM HG medium (1-26F03-I BioConcept Ltd., Allschwil, Switzerland) supplemented with 10% fetal bovine serum (FBS), 2 mM L-Glutamine, and Penicillin-Streptomycin (10,000 IU/mL penicillin, 10 mg/mL streptomycin; 4-01F00-H BioConcept Ltd., Allschwil, Switzerland). Cell cultures were maintained at 37 °C in a humified environment with 95% air and 5% CO_2_.

### 2.6. Cell Viability Assay

To assess the cytotoxic effects of THPs vapors and 1R6F smoke on eukaryotic cells, the NRU assay was performed on MRC-5 cells following a precedent described [29]. A suspension of MRC-5 cells (300 μL) was seeded into 24-well Transwell inserts at a density of 1.5 × 10^5^ cells per well and incubated overnight. Following incubation, the apical medium was removed, and the inserts were relocated to exposure chambers containing 25 mL of DMEM HG to facilitate smoke/vapor ALI exposure. After the exposure, each insert was transferred to a new 24-well plate containing 500 μL of fresh medium in the basal compartment and 300 μL in the apical compartment, allowing the cells to recover for 65 ± 2 h. One day prior to the NRU assay, NRU solution (0.05 g/L) was prepared in fresh medium (1:65) containing 20 mM HEPES buffer and incubated at 37 °C in a 5% CO_2_ atmosphere. On the assay day, the solution was filtered, and the culture medium was removed from both compartments of the inserts. The cells were rinsed twice with pre-warmed PBS and then incubated with 500 μL of the NRU solution in the basal compartment and 300 μL in the apical compartment for 3 h at 37 °C in a humidified atmosphere containing 5% CO_2_. After incubation, unincorporated dye was removed by washing with PBS, and the dye retained within the cells was extracted by adding 330 μL of destain solution (50% ethanol, 49% distilled water, 1% glacial acetic acid) to each insert, followed by shaking at 300 rpm for 10 min. The extracts were transferred to a 96-well plate, with 100 μL added to each well, and the optical density was measured at 540 nm using a reference filter at 630 nm. Blank inserts (without cells) were included to account for background staining, and their values were subtracted from each measurement. The color intensity was determined using a Gen5 Microplate Reader (BioTek Instruments, Winooski, VT, USA). Each sample was analyzed in six replicates, and the results were reported as mean ± SD.

### 2.7. Mutagenicity: AMES Screen

The in vitro mutagenicity of fresh 1R6F smoke and IQOS vapors was evaluated using the Ames test with *Salmonella typhimurium* strain TA98 (Trinova Biochem GmbH, Gießen, Germany) conducted with and without S9 metabolic activation, in accordance with OECD (Organisation for Economic Co-operation and Development) test guideline 471. Daunomycin (6.0 µg/plate) and 2-Aminoanthracene (10.0 µg/plate) were utilized as positive controls for the S9− and S9+ treatments, respectively (ControlChem, Burlington, ON, Canada). Phosphate-buffered saline (PBS) and the S9+ mix served as solvent controls for the S9− and S9+ treatments, respectively. Each concentration of the test vapor or smoke, as well as the positive controls, was tested in triplicate. To prepare the bacterial culture, one STDisc™ (Trinova Biochem GmbH, Gießen, Germania) was inoculated into 25 mL of Nutrient Broth No. 2 (OXOID) in a 50 mL falcon tube and incubated overnight at 37 °C with shaking at 120 rpm. The bacterial suspension was centrifuged at 1800× *g* for 20 min at 4 °C, and the resulting pellet was resuspended in 11 mL of Ca^2+^/Mg^2+^-free PBS. The bacterial suspensions were then exposed to the test aerosol at room temperature, while being shielded from direct light. Specifically, 10 mL of PBS bacterial suspension was placed in an impinger and bubbled with freshly generated smoke (1–5 cigarettes) or aerosol (up to 30 puffs) from the LM1 smoking machine and LM4E vaping machine, respectively. Following each exposure, aliquots of the bacterial suspension were taken from the impingers and directly used for the Ames test, as outlined schematically in Table 1. The suspension was thoroughly mixed and layered onto the top agar on the plates. Plates were rotated and tilted to ensure an even distribution of the top agar layer. Once the agar solidified, the plates were inverted and incubated at 37 °C. After 48 h of incubation, the number of revertant colonies on each plate was counted.

### 2.8. Statistical Analysis

Where applicable, data distribution was assessed using the Shapiro–Wilk test; homoscedasticity was verified using the Brown–Forsythe test. After the normality of the data was assessed, data were analyzed using one-way ANOVA with Bonferroni correction for multiple comparisons. For the agar diffusion test data, Kruskal–Wallis test followed by Dunn’s post hoc test was applied as the homoscedasticity assumption is not satisfied. The significance level was set at 5% (α = 0.05) for all statistical tests. All results and graphs were generated using GraphPad^®^ Prism version 8.2.3 software.

## 3. Results

### 3.1. Antibacterial Activity

#### 3.1.1. Comparative Efficacy of AqExs in Agar Diffusion Test

**Figure 1:**
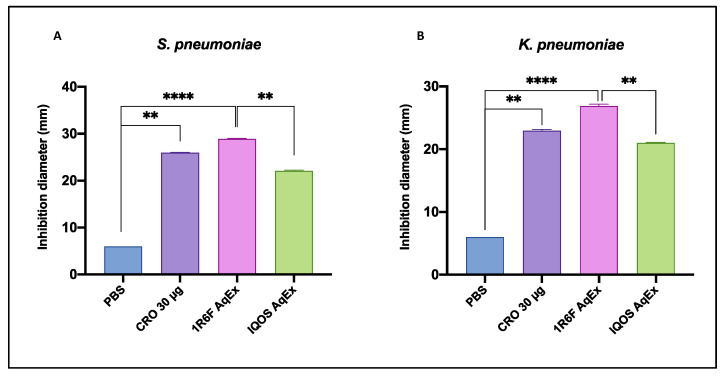
Barplot illustrating inhibition zones measured in the agar diffusion test for (**A**) *S. pneumoniae* and (**B**) *K. pneumoniae*, respectively. Data are reported as means + SD. Statistical significance was indicated as ** *p* < 0.01, **** *p* < 0.0001.

The agar diffusion assay demonstrated significant different antimicrobial effects across the tested solutions, as reported in Table 2 and illustrated in Figure 1. *Streptococcus pneumoniae* showed varying levels of susceptibility to the different AqExs. The IQOS AqEx exhibited an antibacterial effect, with an inhibition zone of 22 ± 0.2 mm, while the 1R6F cigarette smoke AqEx produced the largest inhibition zone, measuring 29 ± 0.2 mm. Similarly, *Klebsiella pneumoniae* demonstrated susceptibility, with inhibition zones of 21 ± 0.1 mm for IQOS AqEx and 27 ± 0.3 mm for 1R6F cigarette smoke AqEx. For comparison, phosphate-buffered saline (PBS), used as a negative control, did not exhibit any inhibition (≤6 mm), whereas the positive control, Ceftriaxone (CRO 30 µg), showed inhibition zones of 26 ± 0.1 mm for *S. pneumoniae* and 23 ± 0.2 mm for *K. pneumoniae*. Comparison between the different conditions tested revealed that 1R6F AqEx produced the most significant zones of inhibition compared with control (PBS) and the other treatments for both *S. pneumoniae* and *K. pneumoniae*. Specifically, the inhibition zones generated by 1R6F AqEx were highly significant (*p* < 0.0001) compared with PBS and significantly larger than those induced by IQOS AqEx (for *S. pneumoniae, p* = 0.002; for *K. pneumonia*, *p* = 0.002). Although clear zones of inhibition were observed for IQOS AqEx in both strains, these did not differ significantly from PBS (*p* > 0.5). Additionally, the inhibition zones produced by 30 µg of CRO were not significantly different from those of either IQOS AqEx or 1R6F AqEx in both strains.

These results indicate that both IQOS aerosol and 1R6F cigarette smoke demonstrated observable antibacterial activity, with the 1R6F cigarette smoke showing the strongest inhibitory effect across both bacterial strains.

#### 3.1.2. Bactericidal and Bacteriostatic Effects Against *S. pneumoniae* and *K. pneumoniae*

MIC and MBC values for *S. pneumoniae* and *K. pneumoniae* were reported in Table 3. For *S. pneumoniae*, IQOS AqEx demonstrated an MIC of 6.25% *v*/*v* and an MBC of 12.5% *v*/*v*, indicating bacteriostatic effects at lower concentrations and bactericidal effects at slightly higher concentrations. Similarly, the 1R6F AqEx showed both MIC and MBC values of 6.25% *v*/*v*, suggesting that it was effective at inhibiting bacterial growth and reducing bacterial viability at the same concentration.

For *K. pneumoniae*, IQOS AqEx also demonstrated an MIC of 6.25% *v*/*v* and an MBC of 12.5% *v*/*v*, mirroring the trend observed for *S. pneumoniae*. In contrast, the 1R6F AqEx exhibited both MIC and MBC values of 6.25% *v*/*v*, indicating a more consistent bactericidal effect even at lower concentrations.

These results further confirm that both IQOS and 1R6F cigarette AqExs possess meaningful antibacterial activity, with 1R6F demonstrating more consistent bactericidal effects across both bacterial strains at lower concentrations.

### 3.2. Cell Viability of MRC-5 Against IQOS vs. 1R6F

**Figure 2:**
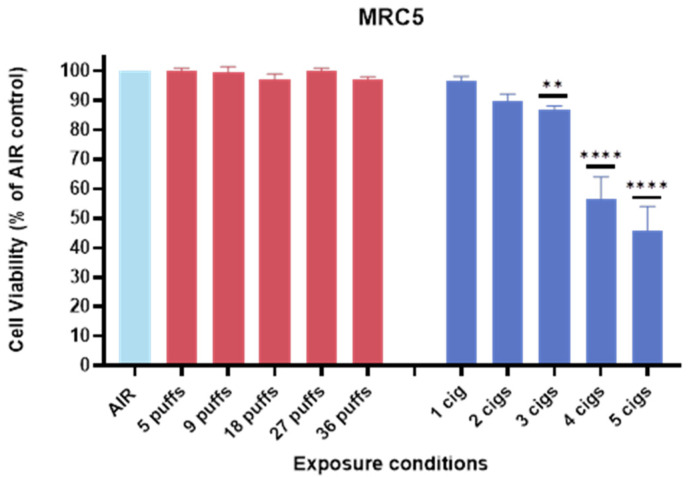
Barplot illustrating MRC-5 cell viability following exposure to THP aerosol (up to 36 puffs) and 1R6F smoke (up to 5 cigarettes). Data are expressed as percentage of the respective air control and displayed as means ± SD, based on 9 replicate wells from triplicate transwell experiments. Statistical significance was indicated as ** *p* < 0.01, **** *p* < 0.0001.

The exposure of MRC5 cells to IQOS did not significantly inhibit cell growth, as indicated in Figure 2. This finding suggested that under the tested conditions, IQOS did not exhibit detectable cytotoxic effects on lung fibroblasts. Following air–liquid interface (ALI) exposure and a 65 h recovery period, the cells maintained viability comparable to the untreated controls, indicating minimal impact on cell proliferation. In contrast, exposure to 1R6F cigarette smoke caused a pronounced, dose-dependent inhibition of cell growth, with marked cytotoxic effects observed at the highest concentrations tested (4–5 cigarettes, Figure 1). This dose–response relationship highlighted the significant harmful impact of conventional cigarette smoke on human lung cells, likely due to the higher levels of toxicants and oxidative stress associated with combustion products. Overall, these results demonstrated that, while traditional cigarette smoke exerted a marked cytotoxic effect on lung fibroblasts, the aerosols from IQOS appeared to be considerably less harmful, at least in terms of short-term cell viability.

### 3.3. Mutagenicity Assessment

To validate the Ames assay, several essential criteria were met, including appropriate responses in positive controls (Control Chems: Sodium Azide, Daunomycin, and 2-Aminoanthracene) and negative controls (solvent and air). For a test substance to be considered mutagenic, the following conditions were required: (1) a twofold increase in revertant colonies compared to the negative control (air), (2) a significantly elevated number of revertants at a minimum of three concentrations of the test substance, (3) a positive, linear dose–response relationship, and (4) reproducible positive results across independent assays. All criteria for a valid Ames test were successfully met. Negative controls (solvent control) remained within expected ranges based on both laboratory experience and literature values [29]. Positive controls were within the acceptable range specified by the manufacturer’s guidelines (Trinova Biochem GmbH, Gießen, Germany). No significant differences were detected between solvent and air controls (*p* > 0.9) under all conditions. However, significant differences (*p* < 0.0001) were observed between these controls and the Chem controls both in the presence and absence of S9 metabolic activation.

**Figure 3:**
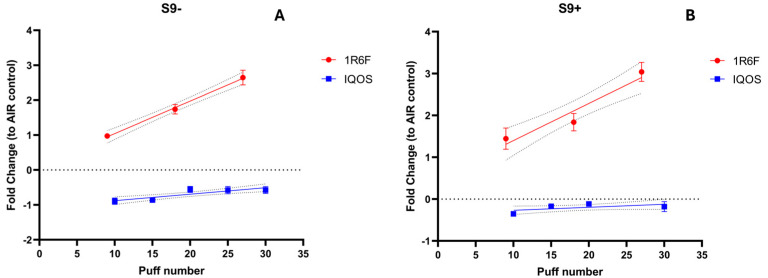
Mutagenicity assessment using the Ames test was conducted on S. typhimurium TA98 without (**A**) and with (**B**) S9 metabolic activation following exposure to 1R6F combustible cigarette smoke (red lines) or IQOS (blue lines). Results were expressed as fold change relative to the air control. The means ± SD of revertant colonies in solvent controls for TA98 during 1R6F experiments were 20.20 ± 2.56 (S9−) and 29.8 ± 13.72 (S9+), whereas for IQOS experiments, they were 5.53 ± 1.95 (S9−) and 6.46 ± 1.59 (S9+). Each data point represents the mean ± SD from triplicate Petri dishes. Dashed lines indicate the 95% confidence interval of the regression line.

As reported in Figure 3, a significant (*p* < 0.05) dose-dependent increase in the number of revertants was observed in the TA98 strain following exposure to 1R6F combustible cigarette smoke, with up to five cigarettes used in the assay, indicating a clear mutagenic effect associated with conventional cigarette smoke. Indeed, a number of revertants without S9 metabolic activation showed approximately a 3.0-fold change relative to the air control (*p* < 0.0001). Similarly, when the Ames assay was conducted with S9 metabolic activation, TA98 showed an increase in revertants of nearly 3.0-fold change relative to the air control (*p* < 0.0001) after exposure to 1R6F cigarette smoke. Conversely, exposure to IQOS aerosol did not result in a statistically significant increase in revertants, even at the highest exposure level (up to 30 puffs), compared to the negative control (both with and without S9 metabolic activation). These findings suggest that while traditional cigarette smoke demonstrates mutagenic potential, the IQOS aerosol does not induce comparable mutagenic effects under the tested conditions.

## 4. Discussion

Respiratory infections caused by opportunistic pathogens such as *Klebsiella pneumoniae* and *Streptococcus pneumoniae* pose significant public health challenges, particularly in individuals with chronic conditions like COPD or in healthcare settings where nosocomial infections are prevalent. *K. pneumoniae* is a leading cause of hospital-acquired pneumonia, while *S. pneumoniae* is a common respiratory commensal that can transition to a pathogen under dysbiotic conditions, causing diseases such as pneumonia, sepsis, and meningitis [30,31]. Both bacteria are influenced by smoking-induced changes in the respiratory tract, including impaired mucociliary clearance, immune modulation, and altered bacterial adherence [32,33].

Nevertheless, our findings demonstrated differences in the effects of conventional cigarette smoke and THP aerosols on microbial dynamics. In terms of cellular toxicity, cigarette smoke induced a marked and dose-dependent reduction in lung fibroblast viability, reflecting the cytotoxic nature of combusted tobacco smoke. In contrast, exposure to THP aerosols did not reduce fibroblast viability, as cell viability levels remained comparable to those of unexposed controls. This finding highlighted a clear toxicological advantage for THPs, as their aerosols lacked the harmful cellular effects associated with conventional cigarettes [4,5]. These results supported their potential role as a harm reduction strategy for individuals unable to quit smoking [34,35,36].

The mutagenic potential, evaluated via the Ames test, further emphasized the divergence between the two products. Conventional cigarette smoke exhibited a strong mutagenic effect, associated with an elevated carcinogenic risk, whereas THP aerosols showed no detectable mutagenic activity. This suggested that THPs presented a lower long-term risk of carcinogenesis compared to conventional cigarettes [6,7].

However, significant concerns arose regarding the impact of THPs on respiratory microbial communities. We exposed the two bacterial strains to 1R6F AqEx (120 puffs/40 mL) and to IQOS AqEx (120 puffs/40 mL). Both *K. pneumoniae* and *S. pneumoniae* were key members of the respiratory microbiota with dual roles as commensals and opportunistic pathogens. IQOS aerosols demonstrated a bacteriostatic effect on *K. pneumoniae*, potentially disrupting its commensal balance. While the inhibition of bacterial growth might have initially appeared beneficial in reducing pathogenic load, it could destabilize the microbial community and trigger a shift from commensal to pathogenic behavior, increasing the risk of infections [37,38,39]. Similarly, *S. pneumoniae* appeared susceptible to potential disruptions caused by THP aerosols, with implications for respiratory health. Alterations in the balance of this bacterium within the microbiota might have increased susceptibility to pneumococcal diseases, particularly in individuals with underlying conditions or compromised immune systems. These findings underscored the need for further research into the long-term effects of THPs on microbial ecosystems and their implications for respiratory health. Although the number of puffs used to generate AqEx from THPs (120 puffs) and conventional cigarettes (48 puffs) differs, our previous experiments demonstrate that nicotine concentrations in extracts prepared using the same method are comparable: 210 µM for IQOS and 212 µM for 1R6F [40]. The dose of nicotine delivered thus reflects equivalent exposure for both products. Therefore, the bacteriostatic and bactericidal activity observed in the assays could be primarily attributed to the nicotine present in the extracts, as previously reported in other studies. [27,41]. This suggests that substances produced during the combustion process in conventional cigarettes may enhance the bactericidal effects beyond those attributable to nicotine alone.

This study is one of the few to compare heated tobacco products and cigarettes, focusing on their effects on respiratory microbes and lung cells, with a unique emphasis on bacterial implications. While it highlights reduced toxicity in heated products, it also acknowledges limitations that warrant attention in future research. First, its focus on short-term toxicological effects leaves the long-term impacts of THPs on respiratory health and microbial communities largely unexplored. Additionally, while the study demonstrates potential disruptions to the respiratory microbiota, it does not account for broader ecological shifts or cumulative impacts on microbial homeostasis over time. The use of controlled laboratory conditions and a limited selection of bacterial strains, specifically *Streptococcus pneumoniae* and *Klebsiella pneumoniae*, restricts the generalizability of the findings to real-world situations and the broader respiratory microbiota. Furthermore, reliance on in-vitro models, though informative, does not fully capture in vivo responses or systemic health effects, highlighting the need for comprehensive, long-term investigations.

## 5. Conclusions

This study highlights a twofold impact of THPs on respiratory health. From a toxicological point of view, THPs showed clear advantages over conventional cigarettes, demonstrating no cytotoxicity or mutagenic potential under the conditions studied. These findings supported the potential of THPs as a harm reduction strategy for smokers unable to quit, offering a less toxic alternative to conventional cigarettes. Conversely, the observed bacteriostatic effects on *K. pneumoniae* and potential disruptions to *S. pneumoniae* have triggered doubts about the impact of THPs on the respiratory microbiota. Alterations in microbial homeostasis could have facilitated the transition of these commensal bacteria to pathogenic states, increasing the risk of respiratory infections and associated complications. In weighing these findings, THPs should be considered a good option for smoking harm reduction, particularly for heavy smokers who are unable or unwilling to quit smoking. However, they were not risk-free and should not have been regarded as a definitive solution to the health risks associated with tobacco use. The potential microbial dysbiosis associated with THPs necessitated cautious evaluation and further investigation to clarify their long-term health implications. Ultimately, while THPs reduced certain risks compared to conventional cigarettes, the broader health implications—especially regarding the respiratory microbiota—remained uncertain. Any public health recommendations need to balance these factors, emphasizing smoking cessation as the optimal strategy for reducing tobacco-related risks, considering THPs in the context of treatment that leads the smoker to complete smoking cessation.

## Figures and Tables

**Table 1:** Schematic representation of the assay. The example is intended for both S9− and S9+ mix in 2 mL of melted top-agar for tube.

Assay Mix	Components to Add into Melted Agar			
	BAqEx *	Air Control	Solvent Control	Control Chem
S9−	200 μL of BAqEx	200 μL of BAqEx air control	100 μL of PBS + 100 μL of the untreated PBS bacterial suspension	100 μL of controlchem solution + 100 μL of untreated PBS bacterial suspension
S9+	500 μL S9+ mix and 50 μL of BAqEx	500 μL S9+ mix and 50 μL of BAqEx air control	500 μL S9+ mix + 50 μL of the untreated PBS bacterial suspension	500 μL S9+ mix + 100 μL of controlchem solution + 50 μL of the untreated PBS bacterial suspension

* Bacterial aqueous extracts.

**Table 2:** Zones of inhibition measured in mm (diameter) caused by the aqueous extracts (AqExs).

Strain	IQOS AqEx	1R6F AqEx	PBS	CRO 30 µg	*p* Value
*S. pneumoniae*	22 ± 0.2	29 ± 0.2	≤6 *	26 ± 0.1	<0.0001
*K. pneumoniae*	21 ± 0.1	27 ± 0.3	≤6 *	23 ± 0.2	<0.0001

Data are presented as mean ± SD; * ≤6: no susceptibility.

**Table 3:** MIC and MBC values of AqExs expressed as % *v*/*v*.

Strain	IQOS AqEx *		1R6F AqEx *	
	MIC	MBC	MIC	MBC
*S. pneumoniae*	6.25	12.5	6.25	6.25
*K. pneumoniae*	6.25	12.5	6.25	6.25

* AqEx: aqueous extract.

## Data Availability

All data generated or analyzed during this study are included in this published article.

## List of Abbreviations

ALI	air–liquid interface
AqExs	aqueous extracts
BAqEx	Bacterial aqueous extracts
COPD	chronic obstructive pulmonary disease
CRO	Ceftriaxone
MBC	Minimal bactericidal concentration
MIC	Minimal inhibitory concentration
THPs	tobacco heating products
